# *In Vitro* Induction of Regulatory CD4^+^CD8α^+^ T Cells by TGF-β, IL-7 and IFN-γ

**DOI:** 10.1371/journal.pone.0067821

**Published:** 2013-07-03

**Authors:** Luc Van Kaer, Whitney A. S. Rabacal, Holly M. Scott Algood, Vrajesh V. Parekh, Danyvid Olivares-Villagómez

**Affiliations:** 1 Department of Pathology, Microbiology and Immunology, Vanderbilt University School of Medicine, Nashville, Tennessee, United States of America; 2 Veterans Affairs Tennessee Valley Healthcare System, Nashville, Tennessee, United States of America; 3 Department of Medicine, Vanderbilt University School of Medicine, Nashville, Tennessee, United States of America; Uniform Services University of the Health Sciences, United States of America

## Abstract

*In vitro* CD4^+^ T cell differentiation systems have made important contributions to understanding the mechanisms underlying the differentiation of naive CD4^+^ T cells into effector cells with distinct biological functions. Mature CD4^+^ T cells expressing CD8αα homodimers are primarily found in the intestinal mucosa of men and mice, and to a lesser extent in other tissues such as peripheral blood. Although CD4^+^CD8α^+^ T cells are easily identified, very little is known about their development and immunological functions. It has been reported, however, that CD4^+^CD8α^+^ T cells possess regulatory properties. In this report, we present a novel *in vitro* differentiation system where CD4^+^ T cells are stimulated to become CD4^+^CD8α^+^ T cells in the presence of TGF-β, IL-7 and IFN-γ, resulting in cells with very similar features as CD4^+^CD8α^+^ intraepithelial lymphocytes. This novel *in vitro* differentiation culture should provide a powerful and tractable tool for dissecting the differentiation and biological functions of CD4^+^CD8α^+^ T cells.

## Introduction

CD4^+^ T cells constitute an important lymphocyte population of the immune system. One of the key features of CD4^+^ T cells is their capacity to differentiate into distinct cellular subtypes with specialized immunological functions. Analysis of the mechanisms underlying CD4^+^ T cell differentiation is of key relevance to understand how immune responses are elicited, controlled and in some cases result in aberrant and unwanted reactions, causing autoimmune and inflammatory disorders.

It was originally believed that, outside of the thymus, CD4^+^ T cells exclude expression of CD8α and β chains. However, more than 15 years ago, several groups identified a population of CD4^+^ T cells co-expressing CD8α-homodimers, which primarily reside in the intestinal intraepithelial lymphocyte (IEL) compartment in mice [1]–[3]. CD4^+^CD8α^+^ IEL derive from mature CD4^+^ T cells reaching the IEL compartment, and these cells most likely represent antigen-experienced lymphocytes with a partially activated phenotype [4]. CD4^+^CD8α^+^ T cells are also found in humans, in association with the intestinal mucosa [5], [6], peripheral blood [7], and tumors [8].

Despite the prevalence of CD4^+^CD8α^+^ T cells in different organs and tissues, very little is known about the maturation of CD4^+^ T cells into CD4^+^CD8α^+^ T cells. Here, we present an *in vitro* differentiation system in which splenic CD4^+^ T cells are skewed towards the CD4^+^CD8α^+^ phenotype. We believe this system will serve as a powerful tool for understanding CD4^+^CD8α^+^ T cell differentiation and the roles these cells play in immune responses.

## Results

### TGF-β, IL-7 and IFN-γ Play a Critical Role in the Generation of CD4^+^CD8α^+^ T Cells

**Figure 1:**
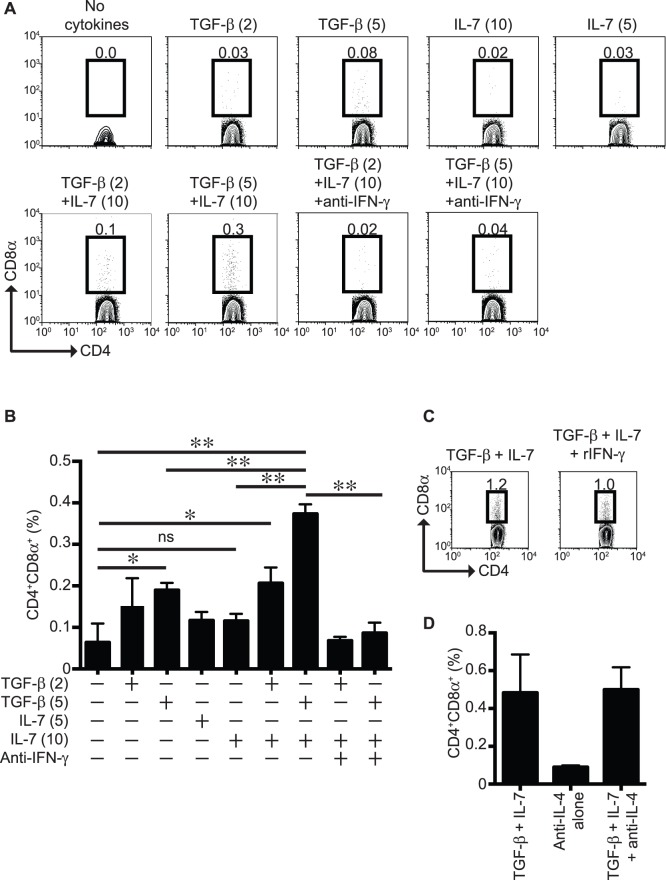
TGF-β, IL-7 and IFN-γ promote the expression of CD8α in CD4^+^ T cells. Total CD4^+^ T cells were stimulated with anti-CD3/CD28 antibodies for four days in the presence or absence of the indicated cytokines and blocking antibodies. (**A**) Representative dot-plots. Cells were gated on live cells by FSC and SSC profile and 7AAD exclusion. Using an anti-CD8β-fluorochrome-coupled antibody, a dump channel served to eliminate cells with unspecific background staining. TGF-β (2): 2 ng/ml; TGF-β (5): 5 ng/ml; IL-7 (5): 5 ng/ml; IL-7 (10): 10 ng/ml. (**B**) Summary of the data presented in (**A**). *P<0.01; **P<0.0001 using one-way ANOVA analysis. No statistical significance was observed between the no cytokine and the TGF-β (2) and IL-7 (5) groups. (**C**) Similar experiment as in (**A**), with inclusion of rIFN-γ in the cultures (right panel). (**D**) Similar experiment as in (**A**), with inclusion of anti-IL-4 antibodies in the cultures. Data is representative of more than 3 independent experiments, n = 3 mice per group.

We have shown that a small fraction of spleen-derived CD4^+^ T cells upregulate CD8α after polyclonal stimulation primarily under Th17-differentiation conditions [9]. Moreover, Konkel et al. demonstrated that the proportion of CD4^+^ T cells expressing CD8α increases in the presence of TGF-β [10]. Consistent with these previous publications, we observed that polyclonal stimulation of CD4^+^ T cells with anti-CD3 and -CD28 antibodies in the presence of 5 ng/ml of TGF-β induced expression of CD8α above background in approximately 0.2% of the total CD4^+^ T cells (Figure 1A and 1B). Because CD4^+^CD8α^+^ T cells represent a considerable fraction of the total CD4^+^ T cells within the IEL compartment, we investigated whether cytokines that are found in the epithelium may promote CD8α expression. IL-7 is expressed by human intestinal epithelial cells [11], its receptor is expressed in mucosal lymphocytes [12], and overexpression of IL-7 in intestinal epithelial cells via the villin promoter increases CD4^+^CD8α^+^ IEL numbers [13]. We therefore decided to investigate whether IL-7 promotes or enhances the expression of CD8α in *in vitro* activated CD4^+^ T cells. Addition of IL-7 alone (5 ng/ml or 10 ng/ml) to the cultures did not increase the proportion of CD4^+^ T cells expressing CD8α beyond background levels (Figure 1A and 1B). However, when both TGF-β (5 ng/ml) and IL-7 (10 ng/ml) were added to the CD4^+^ T cell cultures, we observed a significant increase in CD4^+^ T cells expressing CD8α, reaching levels nearly twice as high as cultures containing high doses of TGF-β alone (Figure 1A and 1B). The percentages of CD4^+^CD8α^+^ T cells induced in cultures containing both TGF-β and IL-7 varied among experiments, falling within a range of 0.3% to 2% of the total numbers of CD4^+^ T cells.

The abundance of IFN-γ-producing lymphoid populations such as CD4^+^ T cells, CD8^+^ T cells and NK cells in the intestinal mucosa suggests that this cytokine is commonly produced in the intestinal epithelium. To determine whether IFN-γ induces the generation of CD4^+^CD8α^+^ T cells *in vitro*, we included IFN-γ in our differentiation cocktail containing TGF-β and IL-7. We observed that the percentages of CD4^+^CD8α^+^ T cells were unaffected by the addition of IFN-γ (Figure 1C). However, addition of anti-IFN-γ antibodies to the differentiation media blunted the expression of CD8α to background levels (Figure 1A and 1B), indicating that the endogenous amounts of IFN-γ produced during activation are sufficient to drive expression of CD8α in CD4^+^ T cells. Blocking CD4^+^CD8α^+^ T cell differentiation by anti-IFN-γ antibodies was specific, because addition of anti-IL-4 antibodies did not affect the generation of CD4^+^CD8α^+^ T cells (Figure 1D).

**Figure 2:**
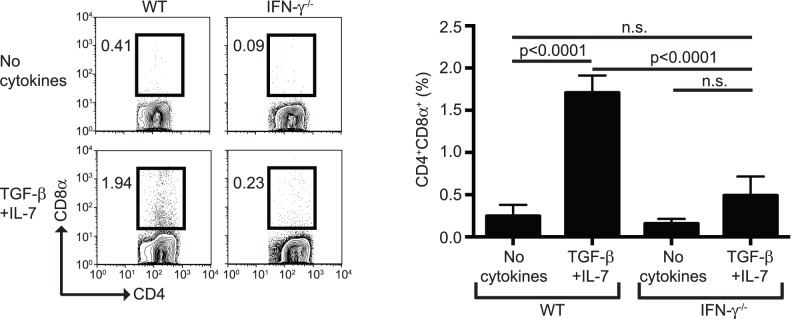
CD4^+^ T cells from IFN-γ^−/−^ mice fail to differentiate into CD4^+^CD8α^+^ T cells. Total CD4^+^ T cells from WT and IFN-γ^−/−^ mice were cultured as in Figure 1A. Left, representative dot-plots. Right, summary of the data presented. Statistical analysis was performed using one-way ANOVA analysis.

To further determine the role of IFN-γ in the differentiαtion of CD4^+^CD8α^+^ T cells, we cultured purified CD4^+^ T cells from IFN-γ^−/−^ mice in the presence of TGF-β and IL-7. As shown in Figure 2, CD4^+^ T cells from IFN-γ^−/−^ mice failed to express CD8α, indicating that this cytokine has a key role in the differentiation of CD4^+^CD8α^+^ T cells.

Collectively, our findings indicate a critical role for TGF-β, IL-7 and IFN-γ in the *in vitro* differentiation of splenic CD4^+^ T cells to CD4^+^CD8α^+^ T cells, a phenotype that is characteristic of a subset of IEL.

### Expansion of CD4^+^CD8α^+^ T Cells Requires TGF-β and IL-7

**Figure 3:**
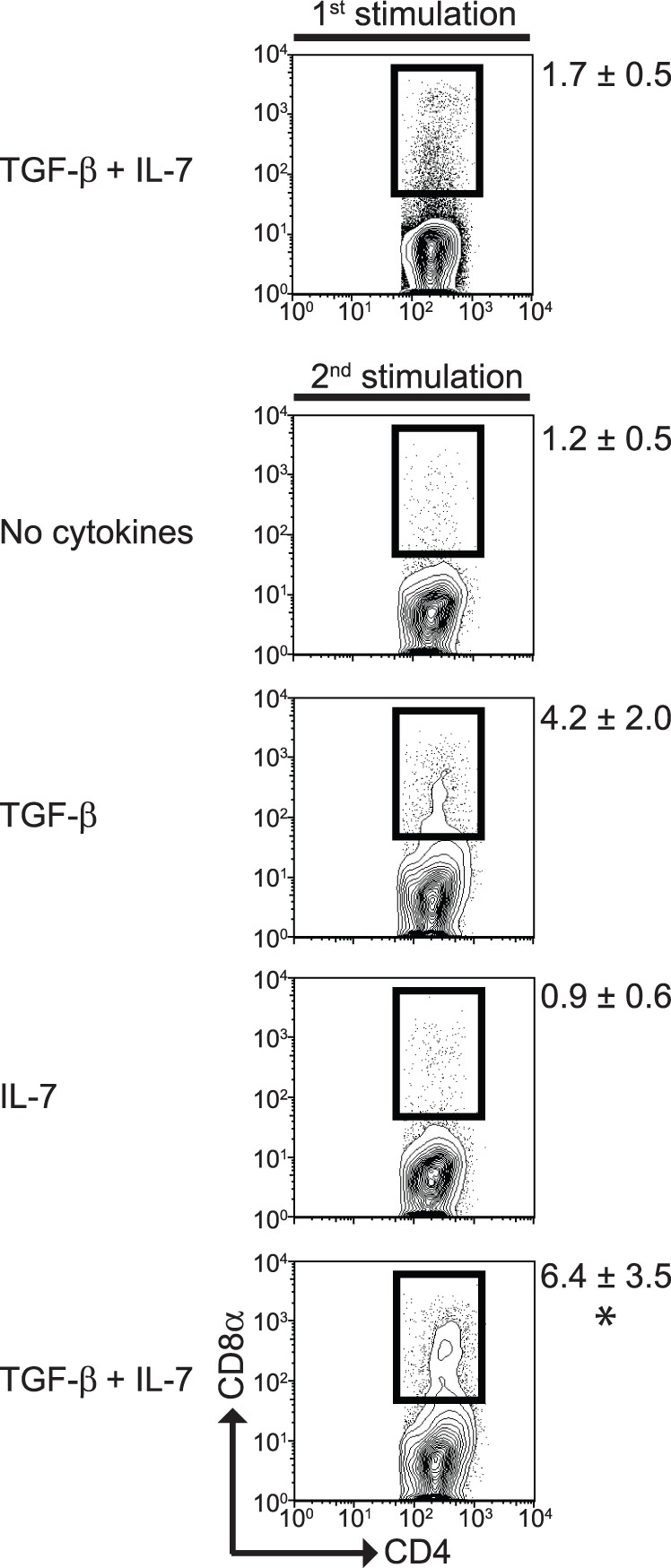
CD4^+^CD8α^+^ T cells expand after secondary stimulation. Total cultures containing CD4^+^CD8α^+^ T cells were stimulated with anti-CD3/CD28 antibodies for a second time in the presence of TGF-β alone, IL-7 alone, TGF-β and IL-7, or in the absence of cytokines. Four days later, cells were analyzed as described in Figure 1A. For comparison purposes, the top panel shows cells after primary stimulation. Data is representative of more than 3 independent experiments. *P<0.05 using one-way ANOVA analysis comparing the no cytokines and TGF-β plus IL-7 groups. No statistical significance was reached when comparing the no cytokines and TGF-β alone groups.

Although TGF-β and IL-7 induced expression of CD8α in CD4^+^ T cells, the average proportion of cells expressing both CD4 and CD8α was around 1% of the total CD4^+^ T cells. In order to determine whether CD4^+^CD8α^+^ T cells could be expanded, we re-stimulated the primary cultures in the presence of TGF-β, IL-7, or both cytokines combined. As shown in Figure 3, a secondary stimulation in the absence of cytokines resulted in similar proportions of CD4^+^CD8α^+^ T cells as observed during primary stimulation. Addition of TGF-β alone during secondary stimulation resulted in a modest increase of CD4^+^CD8α^+^ T cells, but this was not statistically significant. Addition of IL-7 alone during secondary stimulation did not augment the proportions of CD4^+^CD8α^+^ T cell differentiation. However, when both of these cytokines were added during the secondary stimulation we found a substantial increase in the proportion of CD4^+^ cells expressing CD8α, reaching 4 to 10% of the total CD4^+^ T cells. These results indicate that CD4^+^CD8α^+^ T cells can be induced and expanded in our *in vitro* culture conditions, in the presence of TGF-β and IL-7.

### Vitamin D Fails to Promote CD4^+^CD8α^+^ T Cell Differentiation

**Figure 4:**
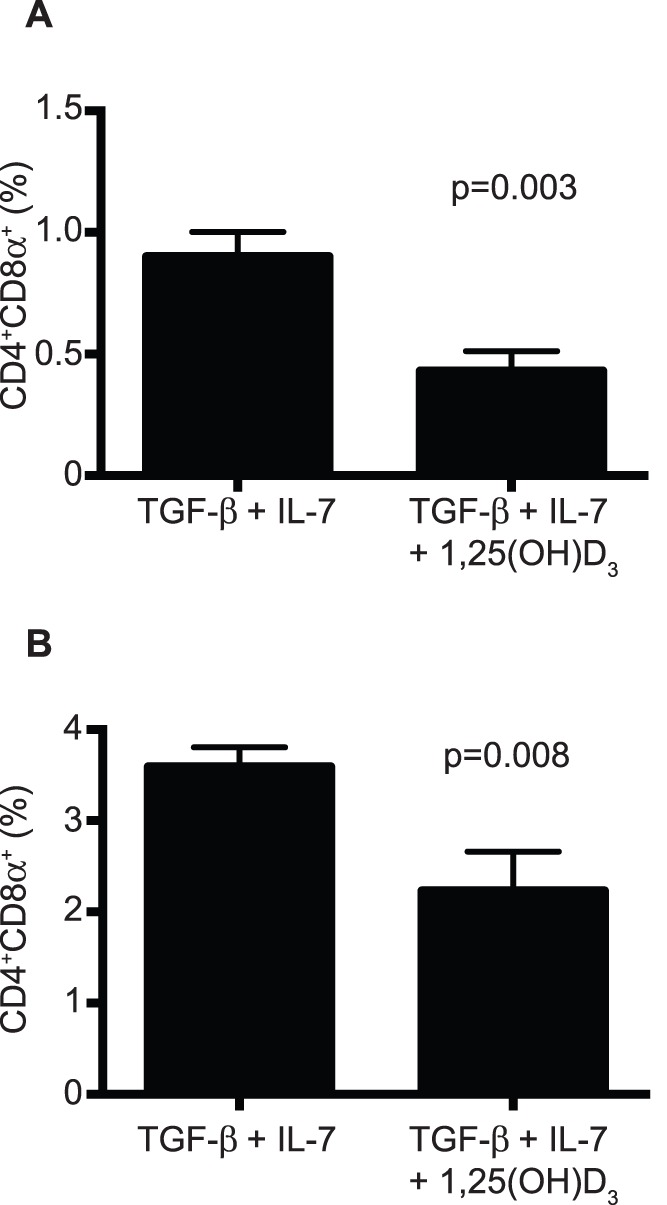
CD4^+^CD8α^+^ T cell differentiation in the presence of vitamin D. The active form of vitamin D, 1,25(OH)D_2_, (50 nM) was added to the differentiation cultures during primary (**A**) or secondary (**B**) stimulation. Data is representative of 2 independent experiments, n = 3.

Mice lacking expression of the vitamin D receptor have a substantial defect in the total number of CD4^+^CD8α^+^ IEL, suggesting that vitamin D has a critical role in the development and/or maintenance of CD4^+^CD8α^+^ IEL [14]. Thus, we determined whether addition of the active form of vitamin D, 1,25(OH)D_3_, facilitated CD4^+^CD8α^+^ T cell differentiation. Surprisingly, the presence of 1,25(OH)D_3_ during primary stimulation reduced the proportions of CD4^+^CD8α^+^ T cells nearly by half (Figure 4A). This reduction was also observed during secondary stimulation (Figure 4B). Thus, at least in the *in vitro* differentiation cultures, vitamin D has a negative effect on the development of CD4^+^CD8α^+^ T cells.

### Cytokine Profile of in vitro Generated CD4^+^CD8α^+^ T Cells

**Figure 5:**
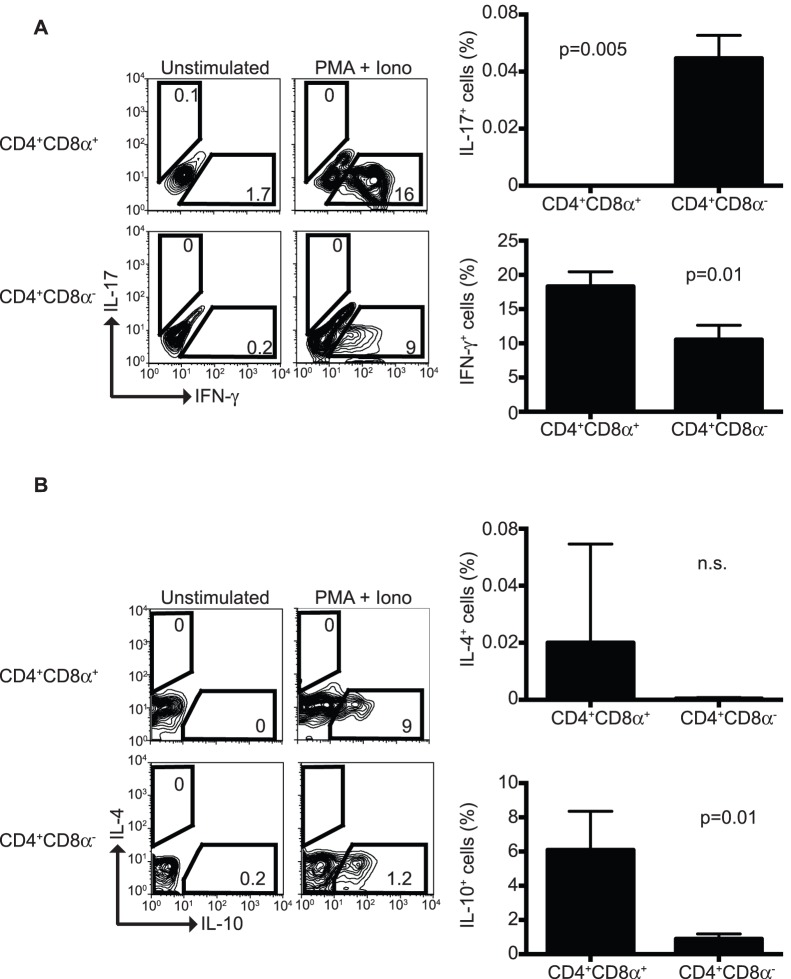
Cytokine profile of differentiated CD4^+^CD8α^+^ T cells. IFN-γ/IL-7 (**A**), and IL-4/IL-10 (**B**) production was determined in differentiated CD4^+^CD8α^+^ and CD4^+^CD8α^−^ T cells after stimulation with PMA/ionomycin followed by intracellular staining. Dot plots are representative data and bar graphs are the summary of the results. Data is representative of at least 2 independent experiments, n = 3.

Previous reports have shown that CD4^+^CD8α^+^ IEL express IFN-γ and IL-10, but lack production of IL-4 [15]. We investigated whether the cytokine profile of *in vitro* generated CD4^+^CD8α^+^ T cells resembles that of *bona fide* CD4^+^CD8α^+^ IEL. In agreement with previous publications, *in vitro* generated CD4^+^CD8α^+^ T cells produced IFN-γ and IL-10 but lacked expression of IL-4 (Figure 5A and 5B). Moreover, we failed to observe production of IL-17, a relevant cytokine in mucosal responses, by CD4^+^CD8α^+^ T cells. However, IL-17 was produced, albeit in very low quantities, by CD4^+^CD8α^−^ T cells generated in the same differentiation cultures. The low levels of IL-17 production observed in CD4^+^CD8α^−^ T cells may indicate lack of other factors that sustain IL-17 production such as IL-6 and IL-23 in the differentiation system. CD4^+^CD8α^−^ T cells also produced IFN-γ, but at lower levels than CD4^+^CD8α^+^ cells (Figure 5A). Thus, these results indicate that our *in vitro* differentiation cultures generate CD4^+^CD8α^+^ T cells with similar cytokine profile as *bona fide* CD4^+^CD8α^+^ IEL, and that the culture conditions have distinctive effects on CD8α^+^ and CD8α^−^ cells.

**Figure 6:**
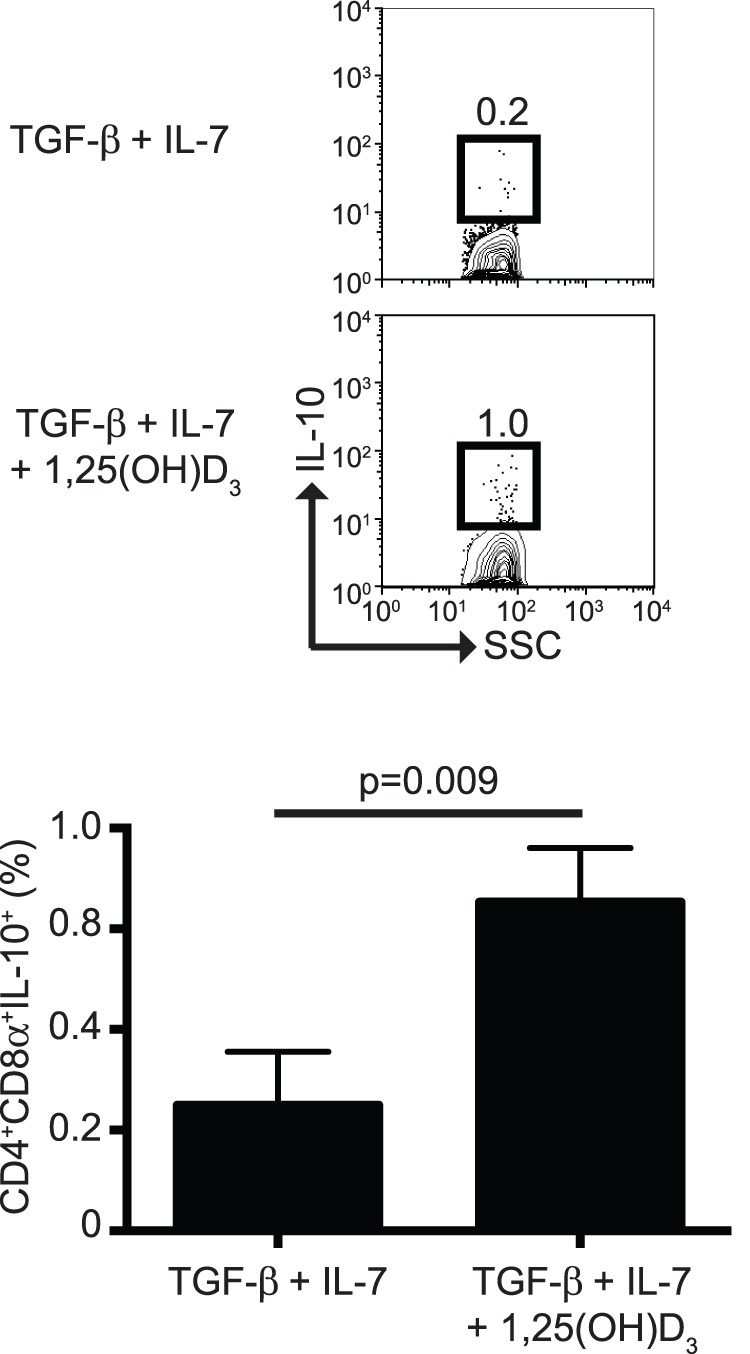
Vitamin D promotes the production of IL-10 by CD4^+^CD8α^+^ T cells. IL-10 production was determined as in Figure 5B during primary stimulation. Dot plots show representative results. Graph represents the summary of the results. Data is representative of at least 2 independent experiments, n = 3.

Although 1,25(OH)D_3_ did not improve the generation of CD4^+^CD8α^+^ T cells (Figure 4A and 4B), we observed that addition of 1,25(OH)D_3_ increased the percentage of IL-10-producing CD4^+^CD8α^+^ T cells (Figure 6), suggesting an important role of vitamin D in IL-10 production capacity. These results are in agreement with previous observations where 1,25(OH)D_3_ increased IL-10 production in human and murine CD4^+^ T cells [16].

### In vitro Generated CD4^+^CD8α^+^ T Cells Express the CD8 Lineage-specific Transcription Factor Runx3

**Figure 7:**
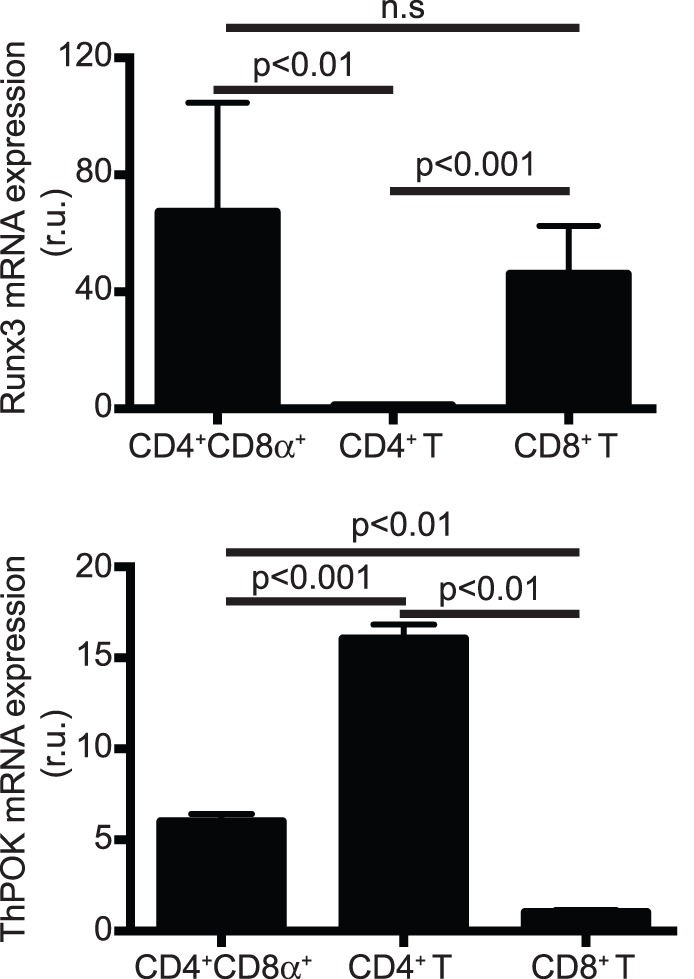
Relative Runx3 and ThPOK mRNA expression. mRNA isolated from FACS-purified CD4^+^CD8α^+^ T cells was reverse-transcribed and the cDNA amplified. (Top graph) Runx3 relative expression was normalized using total naïve CD4^+^ T cell mRNA. (Bottom graph) ThPOK relative expression was normalized using total naïve CD8^+^ T cell mRNA. (n = 3).

Runx3 is a transcription factor implicated in the expression of CD8α [17], which in CD8α^+^ IEL and activated T cells is dependent on the CD8α enhancer E8_I_ [18]. To determine whether expression of CD8α in *in vitro* differentiated CD4^+^CD8α^+^ T cells correlates with Runx3 expression, we determined Runx3 mRNA levels. As shown in Figure 7, CD4^+^CD8α^+^ T cells expressed similar Runx3 mRNA levels than conventional naïve CD8^+^ T cells. Interestingly, we found that CD4^+^CD8α^+^ T cells downregulated expression of the CD4-lineage transcription factor ThPOK, which was not the case for conventional CD4^+^ T cells (Figure 7). These results indicate that differentiation of CD4^+^ T cells towards the CD4^+^CD8α^+^ T cell phenotype correlates with gain of a CD8 lineage-specific transcription factor and partial loss of a CD4 lineage-specific transcription factor, a process that is also observed in CD4^+^CD8α^+^ IEL [19].

### Expression of CTLA-4, Lag-3, NKG2D and PD-1 in in vitro Generated CD4^+^CD8α^+^ T Cells

**Figure 8:**
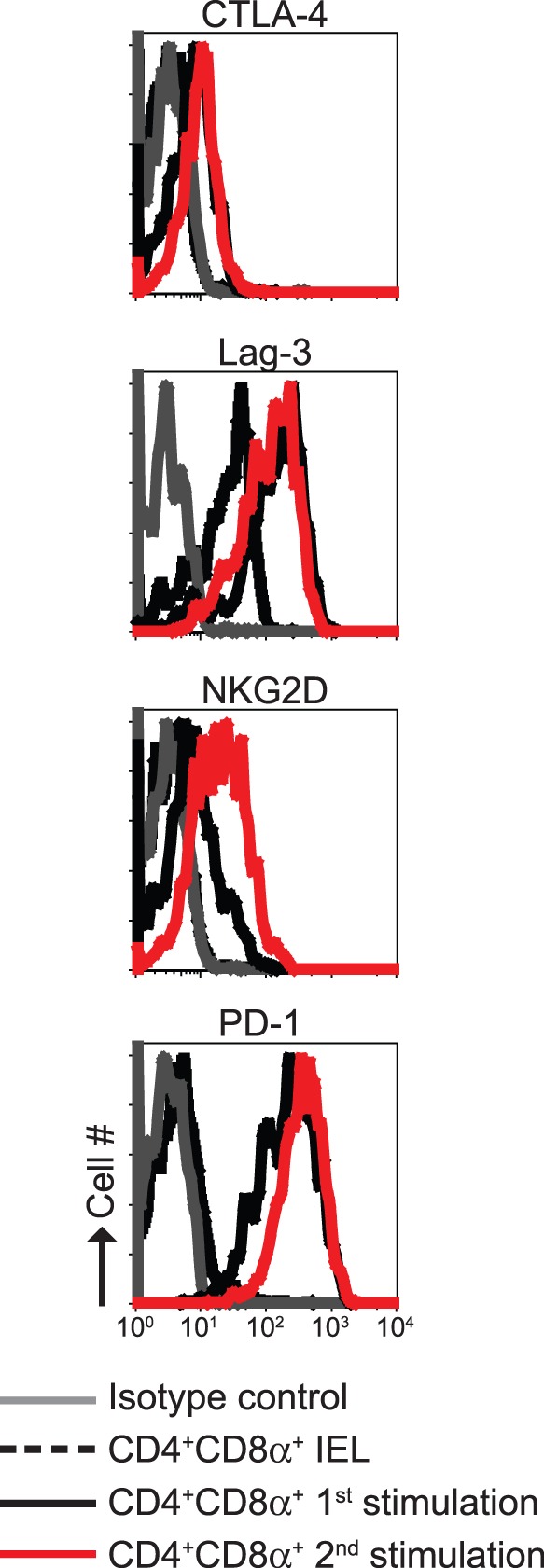
Surface marker expression of *in vitro* generated CD4^+^CD8α^+^ T cells. Expression of the indicated molecules was compared between CD4^+^CD8α^+^ IEL and *in vitro* differentiated CD4^+^CD8α^+^ T cells, after primary and secondary stimulation. Results are representative of 3 independent wild type mice.

The heterogeneity of IEL is not only represented in the expression of TCR αβ or γδ chains, but also in the diversity of expression of other surface proteins (including CD8α). Induced IEL, such as CD4^+^CD8α^+^ cells, can express CTLA-4 [20]; however, CTLA-4 expression was absent in CD4^+^CD8α^+^ IEL isolated from wild type mice, whereas CTLA-4 was upregulated in *in vitro* differentiated CD4^+^CD8α^+^ T cells, during primary and secondary stimulation (Figure 8). Lag-3, a molecule related to CD4 with affinity to MHC class II molecules and involved in regulatory T cell activity [21], was expressed in CD4^+^CD8α^+^ IEL as well as in *in vitro* generated CD4^+^CD8α^+^ T cells (Figure 8). This finding correlates with the potential regulatory properties of CD4^+^CD8α^+^ T cells (see below). We also analyzed NKG2D and PD-1, which are primarily expressed in innate IEL, such as γδ^+^ T cells and TCRαβ^+^CD8αα^+^ IEL [22], [23]. As expected, CD4^+^CD8α^+^ IEL lacked expression of these two molecules, whereas *in vitro* differentiated CD4^+^CD8α^+^ T cells expressed NKG2D and PD-1 after primary and secondary stimulation (Figure 8).

Overall, these results indicate that despite expression of CD8α, differentiated CD4^+^CD8α^+^ T cells differ in the expression of some surface molecules when compared to *bona fide* CD4^+^CD8α^+^ IEL. Thus, our results indicate that other factors present in the intestinal mucosa are necessary for the surface marker profile found in conventional CD4^+^CD8α^+^ IEL.

### Regulatory Properties of in vitro Generated CD4^+^CD8α^+^ T Cells

**Figure 9:**
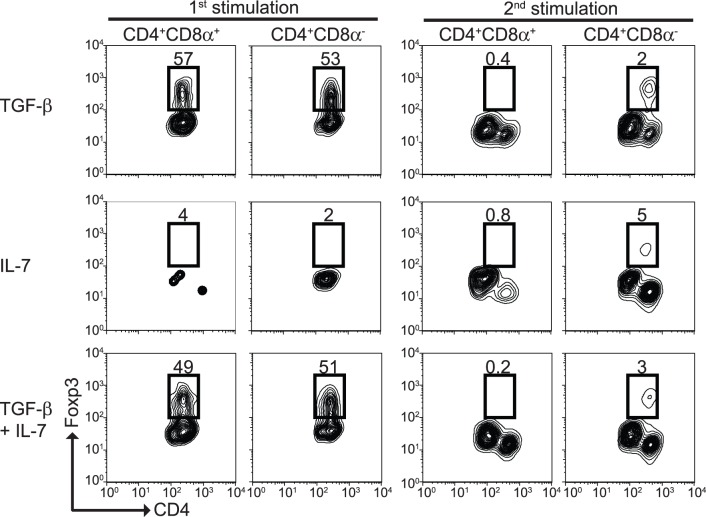
CD4^+^CD8α^+^ T cell Foxp3 expression profile. Expression of Foxp3 was determined in differentiated CD4^+^CD8α^+^ and CD4^+^CD8α^−^ T cells cultured in the presence of the indicated cytokines, either after primary (left columns) or secondary (right columns) stimulation. Data is representative of at least 2 independent experiments, n = 3.

CD4^+^CD8α^+^ IEL are considered to have regulatory properties [15], but lack expression of Foxp3 (data not shown). We found that, during primary stimulation, TGF-β alone or in the presence of IL-7 (but not IL-7 alone) induced expression of Foxp3 in CD4^+^CD8α^+^ and CD4^+^CD8α^−^ T cells (Figure 9, left panels). Interestingly, a secondary stimulation using the same cytokine conditions resulted in almost a complete loss of Foxp3 expression in CD4^+^CD8α^+^ T cells, but not in CD4^+^CD8α^−^ T cells (Figure 9, right panels). Thus, during their differentiation process, CD4^+^CD8α^+^ T cells go through a Foxp3^+^ stage, which is lost after secondary stimulation.

**Figure 10:**
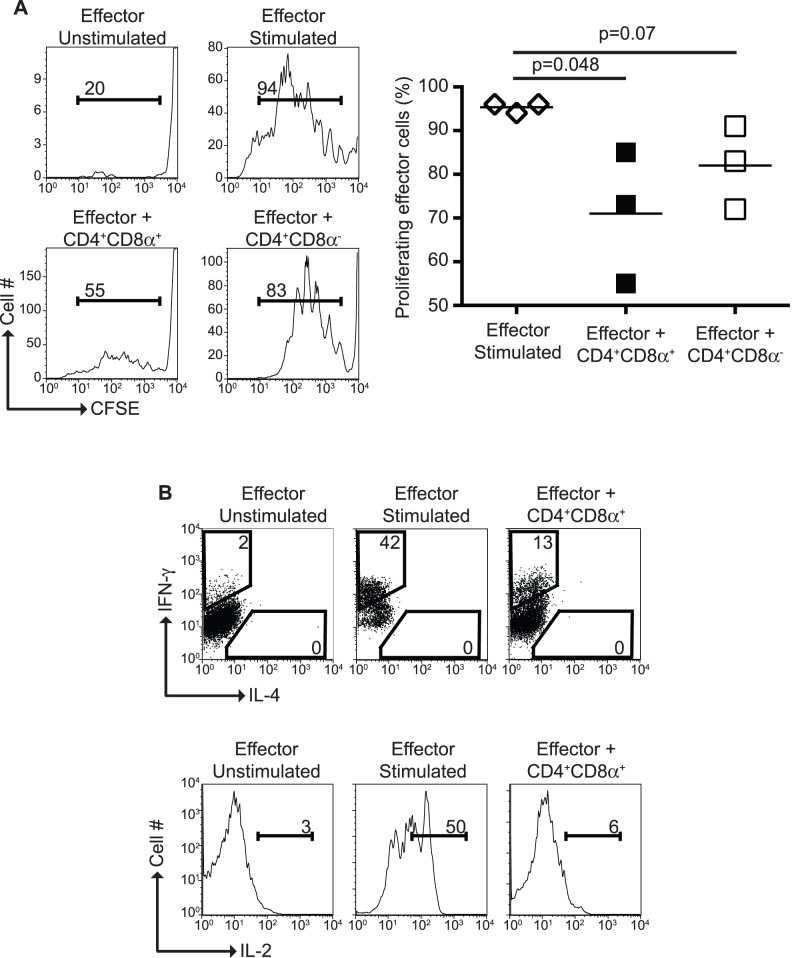
CD4^+^CD8α^+^ T cells exhibit suppressor activity. Purified CD4^+^CD8α^+^ or CD4^+^CD8α^−^ T cells were co-cultured with CFSE-labeled CD4^+^ effector T cells. Six days after culture, effector T cell CFSE dilution (**A**) and IFN-γ, IL-2 and IL-4 production were determined (**B**). Data is representative of at least 2 independent experiments, n = 3..

In order to determine the regulatory potential of *in vitro* differentiated CD4^+^CD8α^+^ T cells, effector T cells derived from CD8α^−/−^ mice (thus preventing expression of CD8α) were cultured alone or in the presence of *in vitro* differentiated CD4^+^CD8α^+^ or CD4^+^CD8α^−^ T cells. As shown in Figure 10A, CD4^+^CD8α^+^ T cells significantly reduced effector CD4^+^ T cell proliferation, and a similar trend (not statistically significant) was also observed when CD4^+^CD8α^−^ T cells were included in the cultures. Control of effector T cell proliferation by CD4^+^CD8α^+^ T cells was accompanied by reduction of effector T cell IFN-γ and IL-2 production (Figure 10B). IL-4 production was not detected in this culture system. These results clearly show that CD4^+^CD8α^+^ T cells possess regulatory potential, a property previously observed in CD4^+^CD8α^+^ IEL [15].

### In vitro Generated CD4^+^CD8α^+^ T Cells and Conventional CD4^+^CD8α^+^ IEL Fail to Reconstitute the Intestinal Mucosa in Adoptive Transfer Experiments

**Figure 11:**
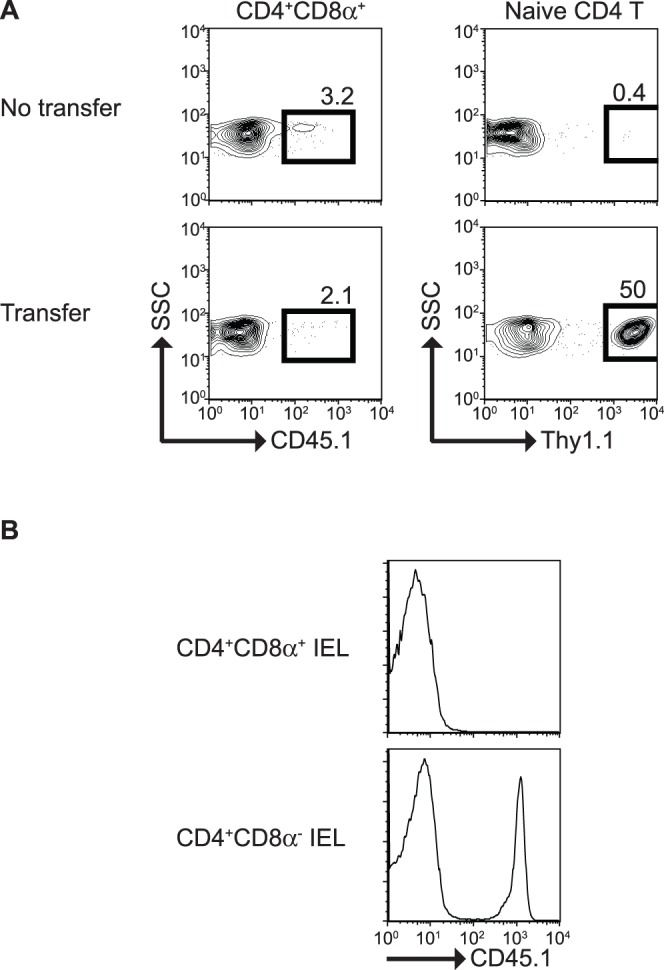
In vitro generated CD4^+^CD8α^+^T cells and CD4^+^CD8α^+^ IEL fail to reconstitute the intestinal epithelium. Purified CD4^+^CD8α^+^ T cells, splenic CD4^+^ T cells (**A**), CD4^+^CD8α^+^ and CD4^+^CD8α^−^ IEL (**B**) were adoptively transferred into RAG-2^−/−^ recipient mice. Four weeks (**A**) or 8 weeks (**B**) post transfer, IEL were isolated and analyzed for reconstitution based on CD45.1^+^ cells. Results show representative analysis of more than three independent experiments. In some instances adoptive transfer was performed i.v. or i.p. with similar results.

Because of prior evidence suggesting that CD4^+^CD8α^+^ T cells possess regulatory functions [15], we adoptively transferred *in vitro* generated CD4^+^CD8α^+^ and naïve splenic CD4^+^ T cells into RAG-2^−/−^ mice. Surprisingly, we were unable to observe reconstitution of CD4^+^CD8α^+^ T cells in the intestinal mucosa in the recipient mice four weeks post-transfer, whereas reconstitution of splenic naïve CD4^+^ T cells was easily detected (Figure 11A). We considered that *in vitro* generated CD4^+^CD8α^+^ T cells needed to express mucosa-specific homing receptors (CCR9 and α4β7) to reach the epithelium, which might not be induced in our culture system. We therefore differentiated CD4^+^CD8α^+^ T cells in the presence of retinoic acid, which is known to induce upregulation of these homing receptors [24]. These CD4^+^CD8α^+^ T cells upregulated CCR9 and α4β7 but still failed to reconstitute the recipient mice (data not shown). These findings suggest that additional signals may be required for migration and survival of *in vitro* generated CD4^+^CD8α^+^ T cells in the intestinal mucosa. Interestingly, in our hands, enriched CD4^+^CD8α^+^ IEL derived from WT animals were unable to reconstitute the mucosal epithelium of recipient RAG-2^−/−^ mice in adoptive transfer experiments, whereas the CD4^+^CD8α^−^ IEL fraction efficiently reconstituted the epithelium (Figure 11B). These results suggest that CD4^+^ T cells acquiring the CD4^+^CD8α^+^ phenotype may lose their capacity to migrate into the mucosal epithelium.

## Discussion

Establishment of *in vitro* culture systems has proven to be an invaluable tool for dissecting important features of CD4^+^ T cell differentiation into distinct helper phenotypes such as Th1, Th2, Th17 and T_reg_. Manipulation of *in vitro* differentiated T cells has also been key for understanding the role of the different CD4^+^ T cell subsets *in vivo*. Here we present a novel *in vitro* differentiation system where a significant fraction of splenic CD4^+^ T cells, in the presence of TGF-β, IL-7 and IFN-γ, is induced to express CD8α. The resulting CD4^+^CD8α^+^ T cell population presents *in vitro* regulatory capacity, characterized by reduction of effector T cell proliferation and production of IL-2 and IFN-γ, presumably mediated by IL-10.

CD4^+^ T cell differentiation into distinct effector populations requires the optimal combination of specific cytokines and, most likely, differentiation of CD4^+^CD8α^+^ T cell must involve an optimal and specific cytokine milieu, composed primarily of those cytokines abundant in the intestinal epithelium. We and other groups [9], [10] have demonstrated that TGF-β promotes expression of CD8αα homodimers by CD4^+^ T cells. However, because TGF-βrepresents a critical modulator of mucosal immune responses and is a key cytokine for the development of other CD4^+^ T effector cells [25], we reasoned that additional cytokines must be involved in the differentiation of CD4^+^CD8α^+^ T cells. Because IEL are characterized by having a “partially” activated phenotype resembling memory T cells [4], one possible cytokine involved in the development of CD4^+^CD8α^+^ T cells is IL-15, which is known to be a selective growth factor for memory T cells [26]. Indeed, IL-15 deficient mice have a reduction in IEL numbers, particularly the TCRαβ^+^CD8αα^+^ subset. Interestingly, however, these mice present with an increase of CD4^+^CD8α^+^ IEL in comparison to wild type animals [27]. Because IL-15 interacts with a receptor complex that includes the common γ chain, which is also used for signaling by IL-2, IL-4, IL-7, IL-9, and IL-21, it is possible that an increase of the CD4^+^CD8α^+^ IEL population in IL-15^−/−^ mice is due to augmentation in the availability of the common γ chain for other cytokine receptors such as IL-7Rα. IL-7Rα^−/−^ mice lack most γδ T cells and have a considerable reduction in the TCRαβ IEL population [28], indicating that IL-7 is an important cytokine involved in the development and/or maintenance of lymphocytes associated with the epithelium. Our results are consistent with these prior studies, and show that the combined effects of IL-7 and TGF-β results in a higher percentage of CD4^+^ T cells expressing CD8α. However, it remains to be determined whether IL-7 induces a differential transcriptional program or promotes proliferation and/or survival of CD4^+^CD8α^+^ T cells.

What is the relevance of an *in vitro* system for the generation of CD4^+^CD8α^+^ T cells? These cells are conspicuously associated with the intestinal epithelium of mice [1]–[3], and have also been identified in the intestinal mucosa of humans [29]. Additionally, CD4^+^CD8α^dim^ T cells have been identified in human peripheral blood [7]. Other studies have reported that human CD4^+^ T cell clones upregulate CD8α chains when cultured in the presence of IL-4 [30]. Despite evidence indicating that CD4^+^CD8α^+^ T cells comprise a bona fide subset of T cells, CD4^+^CD8α^+^ T cells represent a poorly characterized population of mature lymphocytes. Moreover, the role of CD4^+^CD8α^+^ T cells in immune responses is still not completely understood. Das et al. showed that *in vitro* differentiated Th2 cells adoptively transferred into immunodeficient mice were able to reach the mucosa and upregulate CD8α expression [15]. These Th2-derived CD4^+^CD8α^+^ T cells were recovered from the primary recipient mice and subsequently transferred into secondary immunodeficient hosts, where they prevented colitis development induced by pathogenic CD4^+^CD45RB^hi^ T cells [15]. However, a recent publication has challenged these observations by reporting that total CD8αα^+^ IEL were unable to confer protection in a similar adoptive transfer system and, instead, worsened the disease [31]. Further, in patients with ulcerative colitis and Crohn’s disease, the percentage of CD4^+^CD8α^+^ T cells is reduced in the intestinal mucosa, whereas the percentages in peripheral blood are increased [6]. Finally, T cell clones expressing CD4 and CD8α have also been generated from a patient with lepromatous leprosy and from joint fluid of patients with juvenile rheumatoid arthritis [32], [33]. All these reports indicate a putative critical role for CD4^+^CD8α^+^ T cells in certain immune responses.

Because mice deficient in the expression of the vitamin D receptor have a considerable decrease in the numbers and proportions of CD4^+^CD8α^+^ IEL [14], we hypothesized that addition of 1,25(OH)D_3_ would promote CD4^+^CD8α^+^ T cell differentiation. To our surprise, addition of 1,25(OH)D_3_ resulted in a statistically significant decrease in the proportion of CD4^+^CD8α^+^ T cells (Figure 4). Although at present we are unable to explain the observed reduction in T cell differentiation, it is evident that vitamin D is not required for expression of CD8α by CD4^+^ T cells. Yu et al. [14] proposed that vitamin D enhances expression of CCR9 on CD4^+^ T cells and promotes migration of these cells into the intestinal mucosa. Thus, we propose that the *in vivo* generation of CD4^+^CD8α^+^ T cells requires signals for CD4^+^ T cells to migrate into the mucosa (vitamin D, CCR9) and, upon entry into the epithelium, signals that promote expression of CD8αα homodimers (TGF-β, IL-7, and IFN-γ).

*In vitro* differentiated CD4^+^CD8α^+^ T cells possess features that are similar to *bona fide* CD4^+^CD8α^+^ IEL. For example, both types of cells produce IFN-γ and IL-10 but lack production of IL-4, IL-17 and Foxp3 [15], [34]. Furthermore, CD4^+^CD8α^+^ T cells upregulate expression of the CD8 lineage-specific transcription factor Runx3, while downregulating expression of the CD4 lineage-specific transcription factor ThPOK (Figure 7), which is in agreement with recent findings showing that CD4^+^CD8α^+^ IEL differentiation depends on the balance between these two transcription factors [19].

While our *in vitro* differentiation protocol generates CD4^+^CD8α^+^ T cells similar to CD4^+^CD8α^+^ IEL, it remains unclear whether the functional properties of these cells are identical. Nevertheless, our culture system provides a powerful tool to study the differentiation and functional properties of CD4^+^CD8α^+^ T cells.

## Materials and Methods

### Ethics Statement

Mice were maintained in accordance with the Institutional Animal Care and Use Committee (IACUC) at Vanderbilt University. Also, the IACUC at Vanderbilt University specifically approved these studies.

### Mice

C57BL/6, C57BL/6-CD45.1, CD8α^−/−^, IFN-γ^−/−^ and RAG-2^−/−^ mice were purchased from the Jackson laboratories.

### Cell Purification

CD4^+^ T cells were isolated from spleen by enrichment using anti-CD4 beads (Miltenyi) following the manufacturer’s protocol. Purity was usually between 90 to 95%. For effector CD4^+^ T cell isolation, we enriched cells from CD8α^−/−^ mice by depleting B220^+^, DX-5^+^, NK1.1^+^, CD11b^+^, Gr-1^+^, and TCRγδ^+^ cells. Cells were then purified by positive selection of CD62L^+^ cells (Miltenyi). CD4^+^CD62L^+^ T cell purity was >90%. APCs were isolated by depleting TCRαβ^+^ and TCRγδ^+^ cells from total spleen (Miltenyi). *In vitro* differentiated CD4^+^CD8α^+^ T cells were stained with anti-CD4, -CD8α, and -CD8β antibodies and enriched using a FACS Aria sorter. Purity was >95%. IEL were isolated following established procedures [35]. Cells were either used for FACS analysis or for enrichment using a FACS Aria sorter.

### Reagents

Recombinant human TGF-β, IL-7, and recombinant mouse IFN-γ were purchased from R&D, and reconstituted as indicated in the manufacturer’s instructions. TGF-β and IL-7 considerably lost their activity 3 months after reconstitution. To prevent loss of TGF-β activity, reconstituted material was stored in low retention vials. Anti-IFN-γ, -IL-4, -CD3, -CD28 and fluorochrome-coupled anti-CD4, -CD8α, -CD8β, -CD45.1, -CTLA-4, -Foxp3, -IL-4, -IL-2, -IL-17, -IFN-γ, -Lag-3, -NKG2D, -PD-1, and -Thy1.1 antibodies were purchased from BD Pharmingen. The active form of vitamin D, 1,25(OH)D_3_ and retinoic acid were from Sigma. CFSE was purchased from Invitrogen.

### *In vitro* Differentiation of CD4^+^CD8α^+^ T Cells

Purified CD4^+^ T cells were seeded on 24-well plates at a density of 0.5×10^6^ cells/well in a total of 1 ml of RPMI medium supplemented with 10% fetal calf serum, sodium pyruvate and non-essential amino acids. Cells were stimulated with plate-bound anti-CD3 antibodies (5 µg/ml) and soluble anti-CD28 antibodies (2.5 µg/ml). rhTGF-β (2 or 5 ng/ml) [10] and rhIL-7 (5 or 10 ng/ml) [36] were added at the beginning of the culture. Cells were monitored for growth and media consumption. At day 3, media culture presented a slight yellow coloration and cells were blasting. Extensive proliferation and intense yellow media coloration indicated poor CD4^+^CD8α^+^ T cell differentiation, most likely due to loss of TGF-β and/or IL-7 activity. Cells were harvested at day 4 for analysis. For secondary stimulation, cells were washed, seeded and stimulated as described above. Cells were ready for analysis 4 days after secondary stimulation.

In some experiments rIFN-γ (10 ng/ml), 1,25(OH)D_3_ (50 nM), anti-IFN-γ antibodies (10 µg/ml) or anti-IL-4 antibodies (10 µg/ml) were added to the culture media.

### Flow Cytometry

FACS analysis was performed following established protocols [9]. For intracellular staining, cells were washed and stimulated for 4 hours with phorbol-myristate acetate (PMA, 100 ng/ml; Sigma) and ionomycin (1 µM; Sigma) in the presence of GolgiPlug (BD Biosciences). After incubation, cells were washed, stained for surface markers, fixed with 1% paraformaldehyde for 10 minutes, treated with permeabilization buffer (BD Biosciences), and stained for intracellular markers following conventional procedures. Foxp3 intracellular staining was performed following the manufacturer’s instructions (BD biosciences). All samples were acquired using a FACSCalibur Flow System (BD Biosciences) and data were analyzed using FlowJo software (Tree Star).

### *In vitro* T Cell Suppression Assay

*In vitro* T cell suppression assays were performed as described [37]. Briefly, purified CD4^+^CD8α^+^ and CD4^+^CD8α^−^ T cells derived from B6/CD45.1 mice were cultured in the presence of purified CFSE-labeled CD4^+^ effector T cells derived from CD8α^−/−^ mice and irradiated (3000 rads) APCs from spleen, at the following densities: regulator, 3×10^4^ per well; effector, 5×10^4^ per well; APCs, 5×10^4^ per well. Cells were stimulated with 1 µg/ml of soluble anti-CD3 and -CD28 antibodies. CFSE dilution was analyzed 6 days post culture.

### Adoptive Transfer Experiments

Single cell populations were resuspended in PBS and adoptively transferred i.v. or i.p. into 8–10 week old RAG-2^−/−^ recipient mice (∼6×10^4^ to 1×10^5^ per mouse). We did not observe any difference between i.v. or i.p. adoptive transfer.

### Real-time PCR Analysis

RNA was isolated from differentiated and FACS-enriched CD4^+^CD8α^+^ T cells and from magnetically purified naïve CD4^+^ and CD8^+^ splenic T cells, using an RNeasy Qiagen kit. cDNA was synthesized using a reverse transcription kit (Applied Biosciences). For real-time PCR we used the relative gene expression method [38]. Actin served as the normalizer, and cDNA from CD4^+^ or CD8^+^ naïve T cells served as the calibrators. All cDNA samples were analyzed in duplicate using SYBR green (Bio-Rad) as follows: 95**°**C for 3 minutes; 95**°**C for 15 seconds; 60**°**C for 30 seconds; 72**°**C for 20 seconds (40X). Levels of gene expression are relative units based on the comparison of the calibrator with the experimental samples [38]. The primer sequences used were previously described [19].

### Statistical Analysis

Statistical significance between the groups was determined by application of an unpaired two-tailed Student t test. Comparison of three groups or more was determined by application of ordinary one-way ANOVA. A p value <0.05 was considered statistically significant.
